# Early supplemental parenteral nutrition and risk of subsequent enteroatmospheric fistula in high-risk open abdomen patients with persistent enteral nutrition intolerance: a landmark propensity score-matched cohort study

**DOI:** 10.3389/fmed.2026.1845342

**Published:** 2026-05-22

**Authors:** Wenyue Wang, Tian Xie, Chen Chen, Fen Chen, Dongliang Yang, Pengfei Wang, Yousheng Li

**Affiliations:** Department of General Surgery, Shanghai Ninth People’s Hospital, Shanghai Jiao Tong University School of Medicine, Shanghai, China

**Keywords:** acute gastrointestinal injury, critical care, enteral nutrition intolerance, enteroatmospheric fistula, open abdomen, propensity score matching, supplemental parenteral nutrition

## Abstract

**Background:**

High-risk open abdomen (OA) patients with acute gastrointestinal injury (AGI) and persistent enteral nutrition (EN) intolerance are particularly vulnerable to cumulative energy-protein deficit, yet the optimal timing of supplemental parenteral nutrition (SPN) remains uncertain. We evaluated whether earlier SPN initiation was associated with a lower subsequent incidence of enteroatmospheric fistula (EAF) in this population.

**Methods:**

We conducted a single-center retrospective cohort study of adult OA patients admitted to the intensive care unit between January 2019 and December 2024 who had AGI grade II-III, persistent EN intolerance by ICU Day 3, and a modified Nutrition Risk in the Critically Ill score of at least 5. Using ICU Day 7 as the landmark time point, patients who initiated SPN between ICU Days 3 and 7 were classified as the early-SPN group, and those who initiated SPN after ICU Day 7 as the late-SPN group. Patients who never received SPN were intentionally excluded to focus the analysis on the optimal timing among those who definitively required supplementation. One-to-one propensity score matching was performed. The primary outcome was EAF occurring after ICU Day 7.

**Results:**

Among 355 screened patients, 284 met the eligibility criteria, including 110 early-SPN and 174 late-SPN patients; 102 matched pairs were analyzed. During ICU Days 3–7, the early-SPN group received higher energy and protein delivery. EAF occurred in 13/102 patients (12.75%) in the early-SPN group and 31/102 (30.39%) in the late-SPN group (relative risk, 0.42; 95% CI, 0.23–0.75; *p* = 0.002). In an exploratory landmark time-to-event analysis, early SPN was associated with a lower subsequent hazard of EAF (HR, 0.39; 95% CI, 0.21–0.75; log-rank *p* = 0.003). Mechanical ventilation duration was shorter in the early-SPN group (8.27 ± 10.53 vs. 12.00 ± 13.80 days, *p* = 0.031), whereas other secondary outcomes were comparable.

**Conclusion:**

Among high-risk OA patients with AGI and persistent EN intolerance who subsequently required SPN, initiation of SPN between ICU Days 3 and 7 was associated with improved short-term nutritional delivery, shorter mechanical ventilation duration, and a lower subsequent incidence of EAF than later SPN initiation.

## Introduction

Open abdomen (OA) is still an important strategy for selected patients, especially in the presence of severe peritonitis, visceral edema, physiologic exhaustion, or the need for damage control and planned re-exploration (1). However, this approach is also associated with considerable morbidity, such as delayed fascial closure, prolonged catabolism, fluid and protein loss, infectious complications, and enteroatmospheric fistula (EAF), which is one of the most serious complications in this setting (2, 3). EAF is particularly difficult to manage because it complicates wound care, increases the risk of sepsis, worsens malnutrition, prolongs hospital stay, and usually requires long-term multidisciplinary treatment (4–7).

At the same time, acute gastrointestinal injury (AGI) is frequently observed in critically ill patients and is closely associated with poor tolerance to enteral nutrition (EN), systemic inflammation, and unfavorable clinical outcomes (8–11). The ESICM Working Group on Abdominal Problems proposed standardized terminology and grading for AGI, which has helped clinicians recognize AGI as an important part of organ dysfunction in the ICU (8). In patients with AGI grade II-III, enteral feeding often cannot meet early energy and protein requirements, especially when abdominal sepsis, bowel edema, repeated laparotomy, and organ support occur together (8–10, 12). Patients with high nutritional risk, defined by a modified Nutrition Risk in the Critically Ill (mNUTRIC) score ≥5, may be more likely to develop a cumulative early energy-protein deficit (13–16).

The route and timing of nutritional support in critically ill patients remain controversial. Current international guidelines generally recommend early EN when it is feasible, while supplemental parenteral nutrition (SPN) is considered when EN alone is insufficient (17–21). However, the optimal timing for SPN has not been clearly established. In selected high-risk patients, earlier SPN may help reduce cumulative nutritional deficit (19, 22, 23). In contrast, large clinical trials in broader ICU populations have shown neutral or even unfavorable results for routine early parenteral nutrition strategies (21, 24–26). As a result, differences in nutritional practice still exist between guidelines and between different regions (20, 27–32).

In patients with OA, this question may be particularly important. Previous studies in OA have mainly focused on whether EN can be initiated and maintained, whereas evidence regarding when SPN should be introduced after early EN failure remains limited. In clinical practice, SPN is often delayed because of concerns about infectious complications, impaired abdominal healing, or fistula formation. However, in high-risk OA patients with AGI, ongoing abdominal losses, bowel dysfunction, repeated operations, and marked metabolic stress may render prolonged underfeeding especially harmful. Under these circumstances, the clinically relevant question is no longer whether EN should be attempted, but when SPN should be added once early EN has already proven insufficient. Therefore, we conducted a single-center retrospective landmark propensity score-matched cohort study to evaluate, specifically among OA patients who ultimately required SPN, whether initiation between ICU Days 3 and 7 was associated with a lower subsequent incidence of EAF in patients with AGI grade II-III, persistent EN intolerance, and high nutritional risk.

## Materials and methods

### Study design and ethics

We performed a single-center retrospective cohort study in the general surgery ICU of Shanghai Ninth People’s Hospital, Shanghai Jiao Tong University School of Medicine. All consecutive adult patients treated with OA therapy from January 2019 to December 2024 were screened. The study protocol was reviewed and approved by the institutional ethics committee (IRB No. SH9H-2025-T442-1). Because only retrospectively collected and de-identified data were used, informed consent was waived.

### Patient selection

Patients were included if they met all of the following criteria: age ≥18 years, treatment with OA therapy, AGI grade II-III, persistent intolerance to EN on ICU Day 3, and high nutritional risk defined by an mNUTRIC score ≥5. We defined EN intolerance as receipt of less than 60% of the calculated daily energy and protein targets by ICU Day 3. In this study, persistent EN intolerance was operationally defined as failure to achieve at least 60% of both calculated energy and protein targets by ICU Day 3 despite attempted enteral feeding advancement in routine clinical practice. According to standard ICU guidance, the energy target was set at 20–25 kcal/kg/day and the protein target at 1.3–1.5 g/kg/day. For patients with a body mass index (BMI) < 25 kg/m^2^, actual body weight was used; for those with a BMI ≥ 25 kg/m^2^, ideal body weight was applied.

To limit time-related bias, we used ICU Day 7 as the landmark time point. Nutritional exposure was determined at Day 7, and patients who had already developed EAF before that time were not entered into the analytic cohort. We also excluded patients with an mNUTRIC score <5, those discharged or deceased within 72 h, those who never received SPN despite persistent EN intolerance, those who had received PN within 48 h after ICU admission, those with pre-existing chronic PN due to short bowel syndrome, and those with incomplete clinical records. This landmark design was intended to reduce immortal time and outcome-misclassification bias, although all inferences are necessarily conditional on patients remaining alive and EAF-free until the landmark.

### Exposure definition

Patients who started SPN between ICU Days 3 and 7 were assigned to the early SPN (E-SPN) group. Patients who started SPN after ICU Day 7 were assigned to the late SPN (L-SPN) group. Patients who did not receive SPN were not considered part of the comparator group. Since this was a retrospective study, a fully protocolized nutritional strategy could not be replicated, and the exact reasons for delayed SPN initiation may have varied across patients and treating teams. In routine practice, the decision to initiate SPN was made by the treating ICU and surgical teams on the basis of persistent inadequate EN delivery, gastrointestinal function, overall clinical status, and perceived nutritional risk. Specifically, reasons for delaying SPN beyond Day 7 often included concerns regarding potential infectious complications, expectations for spontaneous recovery of gastrointestinal motility, and a clinical preference to maximize enteral feeding attempts before committing to parenteral routes. Daily energy and protein delivery from ICU Days 3 to 7 was collected. Total energy intake included calories from EN, PN, and non-nutritional sources, such as propofol and glucose-containing intravenous infusions.

### Data collection

We extracted baseline demographic, surgical, physiologic, and supportive care data from the electronic medical record. The collected variables included age, sex, body mass index (BMI), mNUTRIC score, APACHE II score, indication for OA, temporary abdominal closure technique, bowel injury, bowel discontinuity, bowel resection, anastomosis, re-exploration, major comorbidities, sepsis on admission, use of mechanical ventilation, and continuous renal replacement therapy (CRRT). Additional outcome-related data, including infection-related complications, laboratory indices, and follow-up information for EAF occurrence and survival, were also extracted from the electronic medical record.

### Outcomes

The primary outcome was EAF occurring after ICU Day 7. EAF was defined as an abnormal communication between the bowel lumen and the atmosphere in the presence of an open abdominal wound. Secondary outcomes were time to primary fascial closure, ICU length of stay, total hospital length of stay, duration of mechanical ventilation, major in-hospital complications, laboratory findings on Day 7, hospital costs, and 90-day survival.

### Statistical analysis

To minimize treatment-selection bias, we performed one-to-one propensity score matching using a multivariable logistic regression model estimating the probability of receiving early SPN. Covariates included in the propensity model were selected *a priori* based on their potential associations with both SPN timing and the risk of EAF, including age, sex, body mass index, mNUTRIC score, APACHE II score, indication for OA, temporary abdominal closure technique, bowel injury, anastomosis, re-exploration, major comorbidities including inflammatory bowel disease, sepsis on admission, and the need for mechanical ventilation or continuous renal replacement therapy. Nearest-neighbor matching without replacement was performed using a caliper width of 0.2 standard deviations of the logit of the propensity score. Covariate balance before and after matching was assessed using standardized mean differences, with an absolute standardized mean difference of less than 0.10 considered indicative of acceptable balance.

Given the potential for time-related bias, a landmark design was used with ICU Day 7 as the exposure assessment time point. Nutritional exposure status was assigned according to SPN initiation before or after the landmark, and patients who developed EAF before ICU Day 7 were excluded from the analytic cohort. For the primary binary analysis, subsequent EAF was compared between groups in the matched cohort. In addition, because post-landmark follow-up times for EAF were available in the updated dataset, we performed an exploratory landmark time-to-event analysis for subsequent EAF. Time zero for this analysis was ICU Day 7, and follow-up continued until EAF occurrence or censoring at the last available follow-up. EAF-free probability after the landmark was estimated using the Kaplan–Meier method and compared using the log-rank test. Cox proportional hazards models were fitted to estimate hazard ratios (HRs) and 95% confidence intervals (CIs). The proportional hazards assumption was assessed using scaled Schoenfeld residuals, including both covariate-specific and global tests. To account for the matched design, robust standard errors clustered by matched pair were used. As sensitivity analyses, we additionally fitted a conditional logistic regression model stratified by matched pair and a further adjusted conditional logistic regression model including mNUTRIC and APACHE II score. Because the extent of missing data was very low (0.8%), we performed a complete-case analysis and did not apply multiple imputation. Although bias due to missing data cannot be completely excluded, the very low proportion of missingness was considered unlikely to materially influence the main findings. Patients with incomplete key clinical records were excluded from the analytic cohort.

Continuous variables are presented as mean ± standard deviation or median (interquartile range), as appropriate, and categorical variables as number (percentage). Between-group comparisons were performed using the Student’s *t*-test or Mann–Whitney U test for continuous variables and the chi-square test or Fisher’s exact test for categorical variables, as appropriate. Kaplan–Meier analysis with the log-rank test was used to compare 90-day survival between groups. All statistical tests were two-sided, and a *p* < 0.05 was considered statistically significant.

## Results

### Patient selection and baseline characteristics

**Figure 1:**
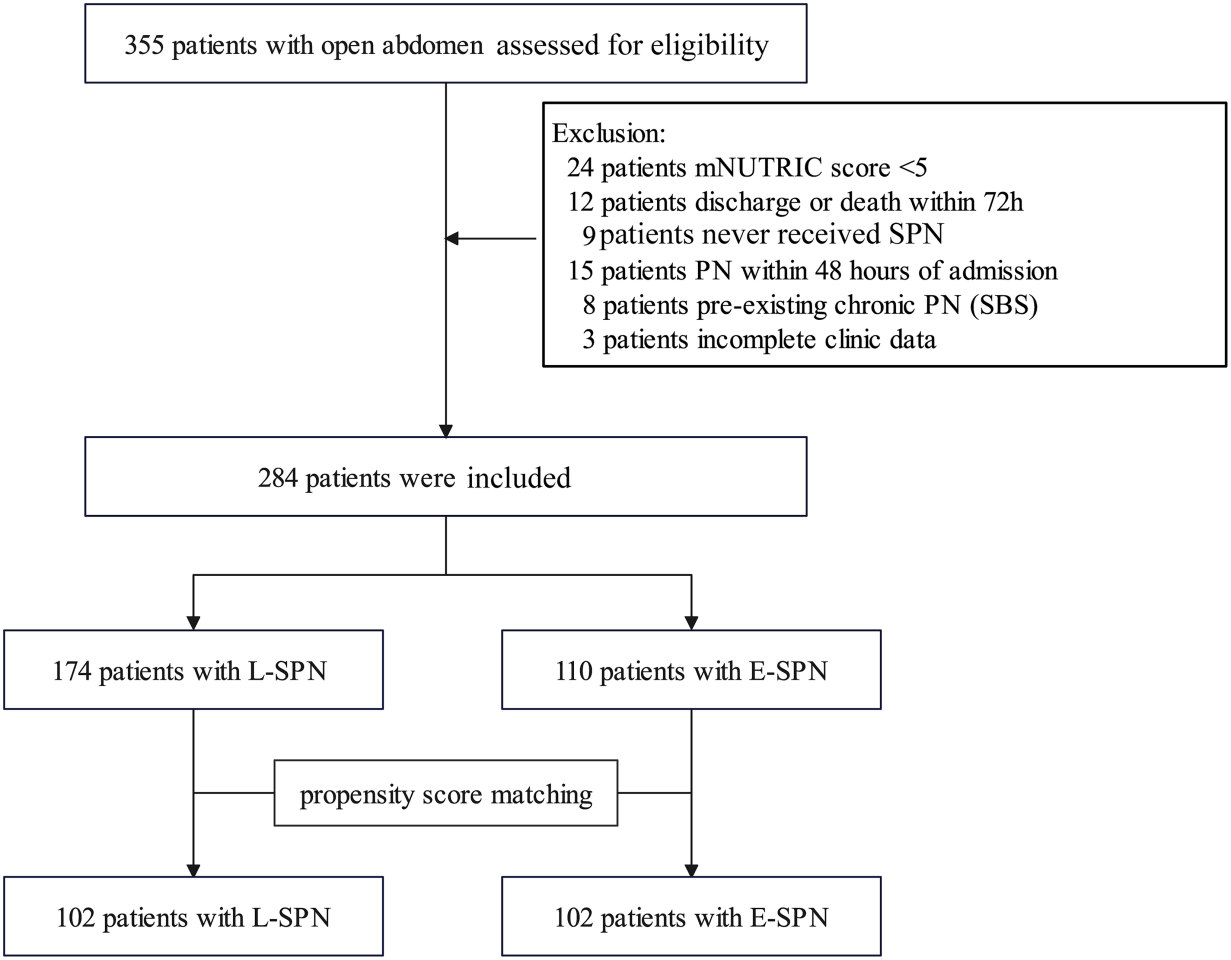
Patient selection and cohort construction. Flow diagram showing patient screening, eligibility assessment, exclusions, exposure classification, and propensity score matching. The analytic cohort was constructed using ICU Day 7 as the landmark time point. E-SPN, early supplemental parenteral nutrition; L-SPN, late supplemental parenteral nutrition; mNUTRIC, modified Nutrition Risk in the Critically Ill; SBS, short bowel syndrome.

Between January 2019 and December 2024, 355 consecutive adult patients treated with OA were screened. After excluding 24 patients with an mNUTRIC score <5, 12 who were discharged or died within 72 h, 9 who never received SPN despite persistent EN intolerance, 15 who received PN within 48 h after ICU admission, 8 with pre-existing chronic PN due to short bowel syndrome, and 3 with incomplete clinical data, 284 patients were included in the final cohort. Among them, 110 patients initiated SPN between ICU Days 3 and 7, whereas 174 initiated SPN after ICU Day 7. After one-to-one propensity score matching, 102 matched pairs were available for analysis (Figure 1).

**Figure 2:**
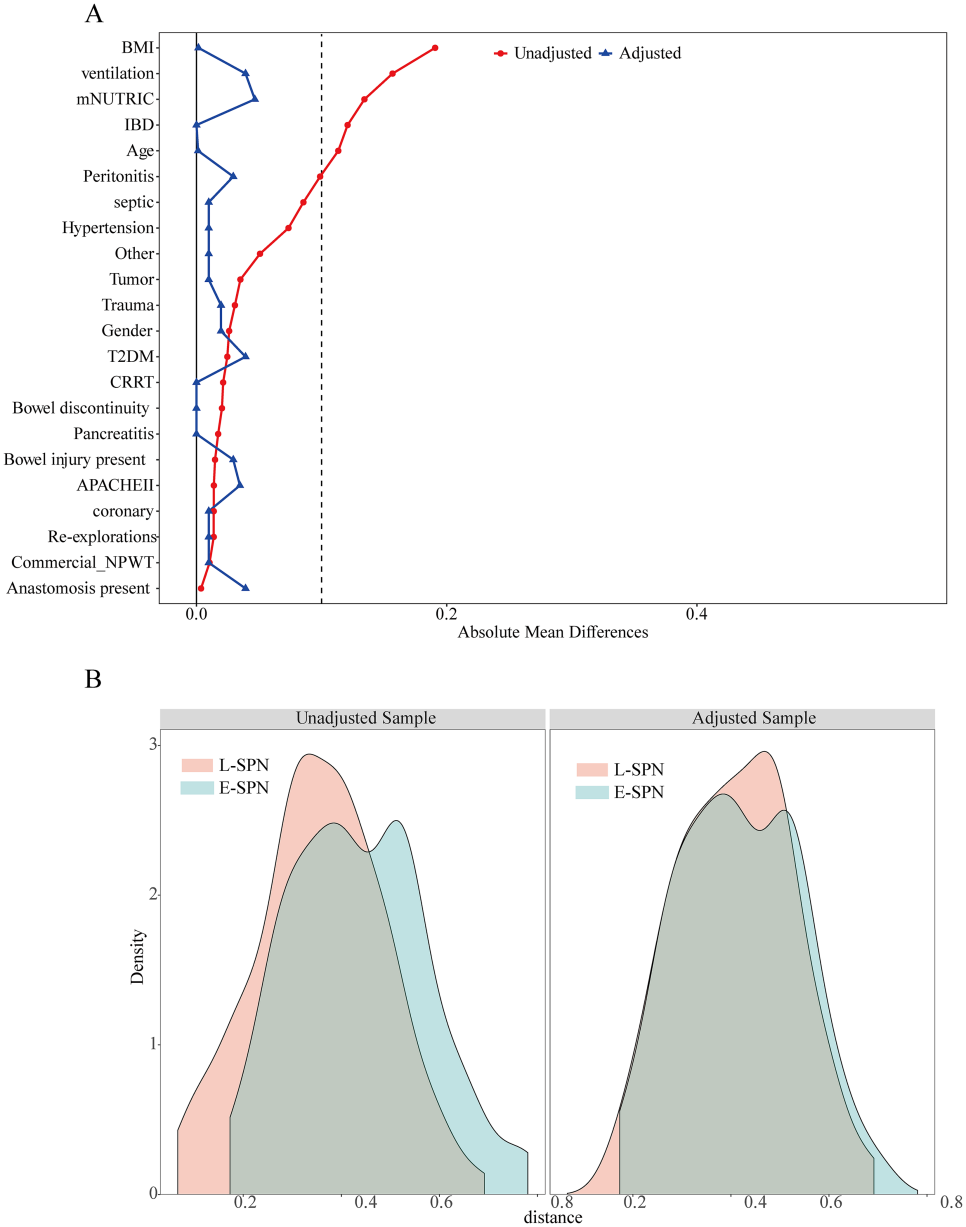
Covariate balance before and after propensity score matching. **(A)** Absolute standardized mean differences for prespecified baseline covariates before and after propensity score matching. The dashed vertical line indicates an absolute standardized mean difference of 0.10. **(B)** Distribution of propensity scores in the E-SPN and L-SPN groups before and after matching. BMI, body mass index; CRRT, continuous renal replacement therapy; E-SPN, early supplemental parenteral nutrition; IBD, inflammatory bowel disease; L-SPN, late supplemental parenteral nutrition; mNUTRIC, modified Nutrition Risk in the Critically Ill; NPWT, negative pressure wound therapy.

Before matching, several clinically meaningful imbalances were observed between the two groups, including a higher proportion of patients requiring mechanical ventilation in the L-SPN group and a higher prevalence of inflammatory bowel disease (Table 1). After matching, baseline demographic, operative, and supportive care characteristics were generally well balanced, and most absolute standardized mean differences were reduced to below 0.10 (Figure 2).

**Table 1:** Baseline demographic and clinical characteristics before and after propensity score matching.

Variables	Before PSM					After PSM				
	Total (*n* = 284)	L-SPN (*n* = 174)	E-SPN (*n* = 110)	*P*	SMD	Total (*n* = 204)	L-SPN (*n* = 102)	E-SPN (*n* = 102)	*P*	SMD
Age	55.93 ± 15.87	55.26 ± 16.31	56.98 ± 15.15	0.375	0.113	57.09 ± 16.08	57.08 ± 16.96	57.10 ± 15.22	0.993	0.001
BMI	20.56 ± 2.90	20.33 ± 2.72	20.93 ± 3.14	0.090	0.191	20.74 ± 2.83	20.74 ± 2.85	20.74 ± 2.83	0.990	−0.002
mNUTRIC	6.00 (5.00, 8.00)	7.00 (5.00, 8.00)	6.00 (5.00, 8.00)	0.303	−0.134	6.00 (5.00, 8.00)	6.00 (5.00, 8.00)	6.00 (5.25, 8.00)	0.646	0.048
APACHE II	15.00 (11.00, 18.00)	15.00 (11.00, 18.00)	16.00 (11.00, 17.75)	0.927	0.014	16.00 (11.00, 18.00)	15.00 (11.00, 18.75)	16.00 (12.50, 18.00)	0.822	0.038
Gender, male, n (%)	202 (71.13)	122 (70.11)	80 (72.73)	0.636	0.059	148 (72.55)	73 (71.57)	75 (73.53)	0.754	0.044
Indication for OA										
Peritonitis	210 (73.94)	122 (70.11)	88 (80.00)	0.064	0.247	157 (76.96)	77 (75.49)	80 (78.43)	0.618	0.072
Pancreatitis	34 (11.97)	22 (12.64)	12 (10.91)	0.661	−0.056	24 (11.76)	12 (11.76)	12 (11.76)	1.000	0.000
Trauma	26 (9.15)	18 (10.34)	8 (7.27)	0.382	−0.118	18 (8.82)	10 (9.80)	8 (7.84)	0.622	−0.073
Others	14 (4.93)	12 (6.90)	2 (1.82)	0.054	−0.380	5 (2.45)	3 (2.94)	2 (1.96)	1.000	−0.071
TAC, n (%)										
Commercial NPWT	260 (91.55)	160 (91.95)	100 (90.91)	0.758	−0.036	189 (92.65)	95 (93.14)	94 (92.16)	0.789	−0.036
Noncommercial apparatus	24 (8.45)	14 (8.05)	10 (9.09)	0.758	0.036	15 (7.35)	7 (6.86)	8 (7.84)	0.789	0.036
OA surgery, n (%)										
Bowel injury present	142 (50)	88 (50.57)	54 (49.09)	0.808	−0.030	105 (51.47)	54 (52.94)	51 (50.00)	0.674	−0.059
Anastomosis present	91 (32.04)	56 (32.18)	35 (31.82)	0.949	−0.008	58 (28.43)	27 (26.47)	31 (30.39)	0.535	0.085
Bowel discontinuity	61 (21.48)	36 (20.69)	25 (22.73)	0.684	0.049	44 (21.57)	22 (21.57)	22 (21.57)	1.000	0.000
Re-explorations	116 (40.85)	72 (41.38)	44 (40.00)	0.818	−0.028	79 (38.73)	40 (39.22)	39 (38.24)	0.886	−0.020
Comorbidities, n (%)										
Hypertension	44 (15.49)	22 (12.64)	22 (20.00)	0.095	0.184	33 (16.18)	17 (16.67)	16 (15.69)	0.849	−0.027
Coronary	26 (9.15)	15 (8.62)	11 (10.00)	0.695	0.046	23 (11.27)	12 (11.76)	11 (10.78)	0.825	−0.032
IBD	52 (18.31)	40 (22.99)	12 (10.91)	0.010	−0.387	24 (11.76)	12 (11.76)	12 (11.76)	1.000	0.000
T2DM	68 (23.94)	40 (22.99)	28 (25.45)	0.635	0.057	46 (22.55)	21 (20.59)	25 (24.51)	0.503	0.091
Tumor	92 (32.39)	54 (31.03)	38 (34.55)	0.538	0.074	69 (33.82)	34 (33.33)	35 (34.31)	0.882	0.021
Sepsis on admission	82 (28.87)	56 (32.18)	26 (23.64)	0.122	−0.201	51 (25)	25 (24.51)	26 (25.49)	0.872	0.022
Ventilation, n (%)	208 (73.24)	138 (79.31)	70 (63.64)	0.004	−0.326	144 (70.59)	74 (72.55)	70 (68.63)	0.539	−0.085
CRRT, n (%)	66 (23.24)	39 (22.41)	27 (24.55)	0.679	0.050	52 (25.49)	26 (25.49)	26 (25.49)	1.000	0.000

Data presentation: Continuous variables are expressed as mean ± standard deviation (SD) or median [interquartile range (IQR)] depending on normality. Categorical variables are expressed as frequency (percentage). Statistical notes: *p*-values were calculated using Student’s *t*-test, Mann–Whitney U test, or Chi-square test as appropriate. SMD denotes standardized mean difference; an absolute SMD <0.10 indicates acceptable covariate balance.

### Nutritional delivery from ICU Day 3 to Day 7

**Figure 3:**
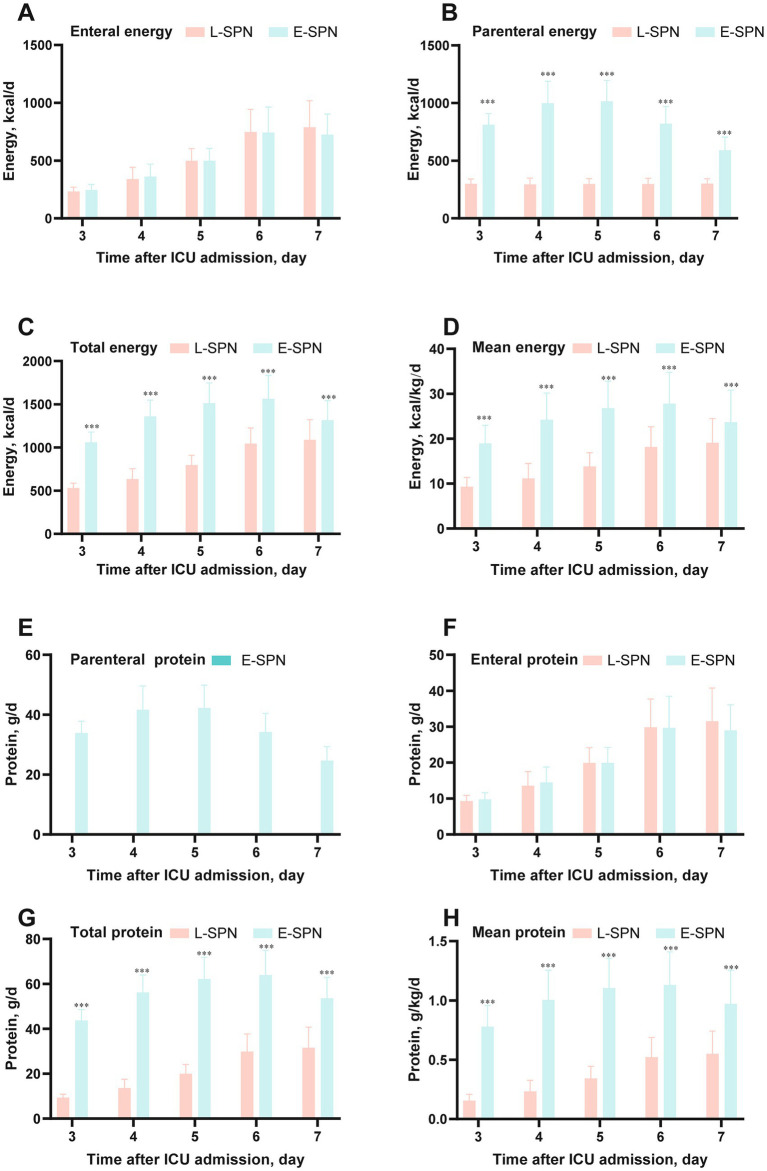
Energy and protein delivery from ICU Day 3 to Day 7 in the matched cohort. Daily nutritional delivery during the exposure window after propensity score matching. **(A–D)** Enteral energy, parenteral energy, total energy, and mean energy delivery, respectively. **(E–H)** Parenteral protein, enteral protein, total protein, and mean protein delivery, respectively. Error bars indicate standard deviation. Between-group comparisons at individual time points are indicated in the figure. E-SPN, early supplemental parenteral nutrition; ICU, intensive care unit; L-SPN, late supplemental parenteral nutrition.

In the matched cohort, patients in the E-SPN group received substantially more nutritional support during the exposure window (Figure 3). Total energy intake was significantly higher in the E-SPN group on each day from ICU Day 3 to Day 7. The mean energy delivery across Days 3–7 was 1361.28 ± 95.70 kcal/day in the E-SPN group, compared with 818.58 ± 69.21 kcal/day in the L-SPN group (*p* < 0.001). A similar pattern was observed for protein delivery. Protein intake on Day 7 was 53.57 ± 9.32 g/day in the E-SPN group and 31.52 ± 9.18 g/day in the L-SPN group, respectively (*p* < 0.001) (Table 2).

**Table 2:** Daily energy and protein delivery from ICU Day 3 to Day 7 in the matched cohort.

Variables	Total (*n* = 204)	L-SPN (*n* = 102)	E-SPN (*n* = 102)	*P*
Total energy				
Day 3	794.52 ± 281.21	530.47 ± 56.68	1058.57 ± 119.36	<0.001
Day 4	997.01 ± 397.13	634.37 ± 119.68	1359.65 ± 189.45	<0.001
Day 5	1153.78 ± 404.12	796.04 ± 114.92	1511.51 ± 235.67	<0.001
Day 6	1303.08 ± 347.53	1044.12 ± 181.72	1562.04 ± 272.28	<0.001
Day 7	1201.24 ± 257.44	1087.88 ± 234.27	1314.61 ± 229.81	<0.001
Mean energy D3- D7	1089.93 ± 285.12	818.58 ± 69.21	1361.28 ± 95.70	<0.001
Total protein				
Day 3	26.49 ± 17.67	9.28 ± 1.54	43.70 ± 4.92	<0.001
Day 4	34.83 ± 22.22	13.60 ± 4.03	56.05 ± 7.85	<0.001
Day 5	41.04 ± 22.49	19.93 ± 4.24	62.15 ± 9.70	<0.001
Day 6	46.87 ± 19.54	29.89 ± 7.83	63.85 ± 11.04	<0.001
Day 7	42.55 ± 14.40	31.52 ± 9.18	53.57 ± 9.32	<0.001
Mean protein D3- D7	38.35 ± 17.92	20.85 ± 2.84	55.86 ± 3.90	<0.001

Data presentation: Values are presented as mean ± SD. Definitions: “Total energy” includes calories from enteral nutrition, parenteral nutrition, and non-nutritional sources (e.g., propofol, glucose infusions). E-SPN was initiated between ICU Day 3 and 7. Statistical significance: *p*-values compare L-SPN vs. E-SPN groups at each time point.

### Primary and secondary outcomes

In the matched cohort, EAF developed in 13 of 102 patients (12.75%) in the E-SPN group and in 31 of 102 patients (30.39%) in the L-SPN group, corresponding to a relative risk of 0.42 (95% CI, 0.23–0.75), an odds ratio of 0.33 (95% CI, 0.16–0.69), and an absolute risk difference of −17.65 percentage points (95% CI, −28.67 to −6.62; *p* = 0.002). The duration of mechanical ventilation was also shorter in the E-SPN group than in the L-SPN group (8.27 ± 10.53 vs. 12.00 ± 13.80 days, *p* = 0.031). By contrast, no statistically significant between-group differences were found in time to primary fascial closure, ICU length of stay, total hospital length of stay, hospital costs, or 90-day survival (Table 3).

**Table 3:** Primary and secondary clinical outcomes in the matched cohort.

Variables	Total (*n* = 204)	L-SPN (*n* = 102)	E-SPN (*n* = 102)	*P*
PFC, days	12.51 ± 2.78	12.73 ± 2.70	12.29 ± 2.86	0.269
LOS	36.26 ± 22.62	39.24 ± 25.26	33.29 ± 19.30	0.061
ICU Days	14.14 ± 6.30	14.83 ± 7.14	13.44 ± 5.28	0.115
Medical expenses, 10,000 yuan	21.01 ± 12.17	19.98 ± 11.00	22.05 ± 13.21	0.225
MV day	10.14 ± 12.39	12.00 ± 13.80	8.27 ± 10.53	**0.031**
Complications, n (%)				
EAF	44 (21.57)	31 (30.39)	13 (12.75)	**0.002**
Cardiovascular complications	23 (11.39)	12 (12.00)	11 (10.78)	0.786
Bleeding	39 (19.12)	22 (21.57)	17 (16.67)	0.373
Pulmonary infections	37 (18.14)	16 (15.69)	21 (20.59)	0.364
Sepsis	33 (16.18)	15 (14.71)	18 (17.65)	0.568
BSI	30 (14.71)	13 (12.75)	17 (16.67)	0.429
Others	22 (10.78)	12 (11.76)	10 (9.80)	0.652

Data presentation: Continuous variables are presented as mean ± SD or median (IQR). Categorical variables are presented as n (%). Outcomes: EAF (primary outcome) was defined as any abnormal communication between the bowel lumen and the atmosphere occurring after ICU Day 7. Bold values indicate statistically significant *p*-values (*p* < 0.05). Medical expenses are reported in 10,000 Chinese Yuan (CNY). BSI, bloodstream infection; CRRT, continuous renal replacement therapy; EAF, enteroatmospheric fistula; ICU, intensive care unit; LOS, length of stay; MV, mechanical ventilation; PFC, primary fascial closure.

**Figure 4:**
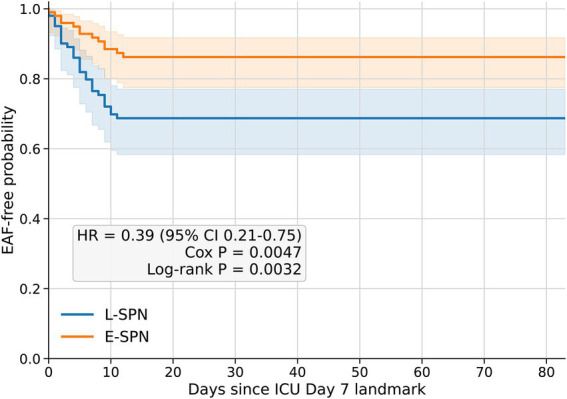
Landmark Kaplan–Meier analysis of post-Day-7 EAF-free probability in the matched cohort. Kaplan–Meier curves showing EAF-free probability after ICU Day 7 in the propensity score-matched cohort. ICU Day 7 was used as the common landmark time point, and patients were followed from the landmark until EAF occurrence or censoring at the last available follow-up. Survival distributions were compared using the log-rank test. The hazard ratio was estimated using a Cox proportional hazards model with robust standard errors clustered by matched pair. EAF, enteroatmospheric fistula; E-SPN, early supplemental parenteral nutrition; ICU, intensive care unit; L-SPN, late supplemental parenteral nutrition.

In an exploratory landmark time-to-event analysis using ICU Day 7 as the common entry time, early SPN was associated with a lower subsequent hazard of EAF than late SPN (HR, 0.39; 95% CI, 0.21–0.75; *p* = 0.0047 by Cox model; log-rank *p* = 0.0032) (Figure 4). Similar findings were observed in a Cox model further adjusted for mNUTRIC and APACHE II score (adjusted HR, 0.35; 95% CI, 0.19–0.67; *p* = 0.0013). In sensitivity analyses, the association between early SPN and a lower subsequent incidence of EAF remained robust in a conditional logistic regression model stratified by matched pair (OR, 0.33; 95% CI, 0.16–0.71; *p* = 0.004) and after further adjustment for mNUTRIC and APACHE II score (OR, 0.31; 95% CI, 0.13–0.73; *p* = 0.007). The proportional hazards assumption was not violated according to scaled Schoenfeld residual testing, either in the exploratory landmark Cox model (global test χ^2^ = 0.140, df = 1, *p* = 0.708) or in the adjusted landmark Cox model including mNUTRIC and APACHE II score (global test χ^2^ = 1.359, df = 3, *p* = 0.715). Additional sensitivity analyses are summarized in Supplementary Table S1.

Laboratory findings on Day 7 were largely similar between the two groups. There were no significant differences in white blood cell count, C-reactive protein, procalcitonin, albumin, prealbumin, creatinine, blood urea nitrogen, calcium, magnesium, or serum iron. Serum phosphate was modestly higher in the E-SPN group than in the L-SPN group (1.24 ± 0.30 vs. 1.12 ± 0.34 mmol/L, *p* = 0.011) (Table 4).

**Table 4:** Laboratory parameters and biomarkers on ICU Day 7 in the matched cohort.

Variables	Total (*n* = 204)	L-SPN (*n* = 102)	E-SPN (*n* = 102)	*P*
Inflammatory indicators at Day 7				
WBC, ×10^9^/L	10.96 ± 9.40	11.97 ± 12.43	9.95 ± 4.59	0.126
CRP, mg/L	77.65 ± 78.87	78.86 ± 79.25	76.44 ± 78.86	0.827
PCT, ng/mL	6.51 ± 13.01	5.29 ± 11.79	7.72 ± 14.07	0.182
Nutritional indicators at ICU Day 7				
Albumin, g/L	34.23 ± 4.56	34.14 ± 4.15	34.32 ± 4.96	0.771
Prealbumin, mg/L	133.08 ± 29.87	131.05 ± 24.92	135.11 ± 34.12	0.333
Kidney function				
CRE, μmol/L	76.96 ± 64.05	77.66 ± 76.16	76.25 ± 49.43	0.876
BUN, mmol/L	9.25 ± 10.37	9.58 ± 9.76	8.91 ± 11.00	0.647
Electrolytes				
Calcium, mmol/L	2.04 ± 0.22	2.03 ± 0.24	2.04 ± 0.20	0.686
Serum Phosphate, mmol/L	1.18 ± 0.33	1.12 ± 0.34	1.24 ± 0.30	**0.011**
Magnesium, mmol/L	0.79 ± 0.19	0.80 ± 0.20	0.79 ± 0.19	0.705
Iron, μmol/L	9.84 ± 5.24	9.83 ± 5.76	9.84 ± 4.69	0.993

Data presentation: Values are presented as mean ± SD. BUN, blood urea nitrogen; CRE, creatinine; CRP, C-reactive protein; PCT, procalcitonin; WBC, white blood cell count. Bold values indicate statistically significant *p*-values (*p* < 0.05).

## Discussion

In this single-center landmark propensity score-matched cohort study of high-risk OA patients with AGI and persistent EN intolerance, earlier initiation of SPN between ICU Days 3 and 7 was associated with a lower subsequent incidence of EAF compared with later initiation after ICU Day 7. Patients in the early SPN group also achieved greater energy and protein delivery during the exposure window and had a shorter duration of mechanical ventilation. This association was further supported in an exploratory post-landmark time-to-event analysis. Importantly, these associations were not accompanied by an apparent increase in infectious complications, as rates of sepsis, bloodstream infection, and inflammatory laboratory markers were broadly comparable between groups. In contrast, no significant differences were observed in time to primary fascial closure, ICU length of stay, total hospital stay, or 90-day survival. Taken together, these findings suggest a potentially clinically relevant association between earlier SPN and selected short-term outcomes among OA patients who ultimately require SPN, but they do not establish benefit across all OA patients or across broader patient-centered outcomes.

The timing of SPN in critically ill patients remains debated, and our findings should be interpreted within the context of patient selection rather than as support for a universal early PN strategy. Contemporary guidelines generally endorse early EN when feasible and recommend considering supplemental PN when EN alone remains insufficient, but they differ in how strongly they support earlier versus later supplementation (17–20). A key issue is that evidence from unselected ICU populations may not be directly applicable to OA patients with AGI, persistent EN intolerance, and high nutritional risk. In this setting, ongoing abdominal losses, bowel dysfunction, repeated operations, and systemic catabolism may render prolonged underfeeding more clinically consequential than in broader ICU cohorts. Our results therefore support the view that the effect of SPN timing is likely context-dependent and may be most relevant in selected patients with clear early nutritional inadequacy. Because the comparator was intentionally restricted to patients who ultimately received SPN, our findings do not address whether SPN itself should be used and cannot be generalized to OA patients who tolerated EN adequately or never required SPN.

This may also help explain why our findings are not fully consistent with some landmark trials. In the EPaNIC study, late PN was associated with faster recovery and fewer complications in a general ICU population (21). However, Heidegger et al. showed that in patients who were unable to achieve energy targets with EN alone by Day 3, SPN initiated between Days 4 and 8 reduced the incidence of nosocomial infection (22). Similarly, Doig et al. reported beneficial effects of early PN in patients with temporary contraindications to early EN (23). Other large studies, including CALORIES, and subsequent meta-analyses did not demonstrate a clear mortality difference between enteral and parenteral nutrition, suggesting that overall nutritional exposure and patient phenotype may be more important than feeding route alone (24–28, 33, 34). In this respect, our cohort was clearly different from an unselected ICU population, as it represented a highly selected subgroup with obvious early EN failure. In such patients, the clinical problem is not whether EN should be attempted, but rather whether ongoing nutritional inadequacy should be tolerated once early EN has already proven insufficient. From this perspective, SPN should be understood as a supplement to failed early EN rather than as a competing feeding strategy.

The pathophysiological features of OA also provide a reasonable explanation for our findings. Patients managed with OA are often in a pronounced hypermetabolic and catabolic state. Ongoing loss of protein-rich fluid, repeated inflammatory insults, multiple abdominal re-explorations, and severe gastrointestinal dysfunction can rapidly lead to cumulative nutritional deficits (1–3). Previous studies in OA have generally supported the importance of early nutritional therapy. A 2024 study of OA patients treated with negative pressure therapy reported that initiation of enteral feeding within 7 days was associated with better overall outcomes, although the study was not designed to specifically evaluate the relationship between caloric delivery and fistula formation (2). Interim findings from the IROA registry also suggested that the occurrence of EAF may be related to both OA duration and baseline nutritional status (3). In fact, our study addresses a somewhat different but clinically practical issue. The question is not whether EN should be attempted, but what should be done when EN still fails to reach 60% of the target by Day 3. Under this condition, SPN should be understood as a supplement to insufficient EN rather than a replacement for it.

It should also be recognized that earlier SPN was not associated with improvement in several broader clinical outcomes, including 90-day survival, ICU length of stay, total hospital stay, or time to primary fascial closure. This pattern is clinically plausible. Compared with mortality or resource utilization, EAF may represent a more proximal and nutrition-sensitive complication in OA patients with persistent EN inadequacy. By contrast, outcomes such as survival, length of stay, and abdominal closure are influenced by multiple interrelated factors, including adequacy of source control, burden of sepsis, severity of organ dysfunction, number of re-operations, and the overall trajectory of recovery. Accordingly, the absence of a measurable survival benefit should not be interpreted as evidence against the clinical relevance of the observed association. Given the substantial morbidity, treatment complexity, and resource burden associated with EAF, a lower subsequent incidence of this complication may still be clinically meaningful.

Several limitations should be considered when interpreting the present findings. First, this was a single-center retrospective study, and as such, generalizability is inherently limited, particularly because patient profiles and OA management patterns may differ across regions and healthcare systems (35). Residual confounding cannot be fully excluded despite the use of both landmark analysis and propensity score matching. Unmeasured factors such as subtle differences in operative decision-making, adequacy of source control, precise surgical strategies for abdominal closure, the specific degree of intra-abdominal contamination, dynamic fluid balance, and clinician-level nutrition practices may have influenced both the timing of SPN initiation and the subsequent risk of EAF. Consequently, residual confounding by indication remains a possibility despite propensity score matching. Second, although the study specifically compared earlier versus later SPN initiation among patients who ultimately required SPN, the reasons for delaying SPN were not fully protocolized and may have reflected heterogeneity in illness trajectory, perceived feeding tolerance, and treating-team preferences. Moreover, the exclusion of patients who never received SPN introduces selection bias, meaning our findings apply only to patients who ultimately received SPN. Additionally, while the landmark design effectively mitigates immortal time bias regarding the timing of SPN exposure, it inherently introduces survivor bias, as only patients who survived and remained EAF-free up to ICU Day 7 were included in the analytic cohort. Third, nutritional targets were estimated rather than guided by indirect calorimetry, and neither nitrogen balance nor abdominal effluent-associated nutrient loss was quantified; therefore, the actual magnitude of cumulative nutritional deficit may have been incompletely captured. Fourth, the sample size, although adequate for the primary analysis, remained limited and underpowered for broader outcomes such as 90-day survival. In addition, although post-landmark follow-up times for EAF were available for exploratory time-to-event analyses, minor inconsistencies across some recorded time variables limited the precision of these analyses; therefore, the Cox results should be interpreted as complementary to, rather than replacing, the primary matched-cohort binary analysis.

## Conclusion

Among high-risk OA patients with AGI and persistent EN intolerance who subsequently required SPN, initiation of SPN between ICU Days 3 and 7 was associated with improved short-term energy and protein delivery, a shorter duration of mechanical ventilation, and a lower subsequent incidence of EAF compared with later SPN initiation. However, given the lack of significant differences in overall mortality and length of stay, the clinical implications of these findings should be interpreted cautiously. These findings support avoidance of prolonged underfeeding in selected OA patients with early EN failure and provide rationale for prospective validation of SPN timing in this high-risk subgroup.

## Data Availability

The datasets presented in this article are not readily available because the datasets generated and analyzed during the current study are not publicly available due to institutional restrictions regarding patient privacy but are available from the corresponding author on reasonable request. Requests to access the datasets should be directed to Yousheng Li, gisurgery@yeah.net.
