# Supplementary material for: Early supplemental parenteral nutrition and risk of subsequent enteroatmospheric fistula in high-risk open abdomen patients with persistent enteral nutrition intolerance: a landmark propensity score-matched cohort study
Source: Front Med (Lausanne). 2026 May 22;13:1845342. doi: 10.3389/fmed.2026.1845342 (PMC13237692; doi:10.3389/fmed.2026.1845342)
Supplement: Supplementary file 1 [file Table_1.docx]

# Supplementary Table S1. Sensitivity analyses for the association between early supplemental parenteral nutrition and subsequent enteroatmospheric fistula in the matched cohort

| Analysis | Model specification | Effect estimate (95% CI) | P value |
| --- | --- | --- | --- |
| Exploratory landmark Cox model | ICU Day 7 landmark analysis with robust standard errors clustered by matched pair | HR 0.39 (0.21-0.75) | 0.0047 |
| Adjusted landmark Cox model | Landmark Cox model additionally adjusted for mNUTRIC and APACHE II score | HR 0.35 (0.19-0.67) | 0.0013 |
| Conditional logistic regression | Model stratified by matched pair | OR 0.33 (0.16-0.71) | 0.004 |
| Adjusted conditional logistic regression | Conditional logistic model stratified by matched pair and additionally adjusted for mNUTRIC and APACHE II score | OR 0.31 (0.13-0.73) | 0.007 |

Data are presented as effect estimates with 95% confidence intervals. HR, hazard ratio; OR, odds ratio; EAF, enteroatmospheric fistula; E-SPN, early supplemental parenteral nutrition.
